# Single Cell Meta-Analysis of Endothelial to Mesenchymal Transition (EndMT) in Glucose Metabolism of the Digestive Diseases

**DOI:** 10.3389/fmolb.2022.866408

**Published:** 2022-06-08

**Authors:** Qiao Zhou, Xin Zhang, Xia Tong, Chuang Tang, Xin Chen, Ling Peng, Xiangen Xia, Lanlan Zhang

**Affiliations:** ^1^ Department of Gastroenterology, West China (Airport) Hospital, Sichuan University/The First People’s Hospital in Shuangliu District, Chengdu, China; ^2^ Division of Pulmonary Diseases, State Key Laboratory of Biotherapy of China, and Department of Respiratory Medicine, West China Hospital of Sichuan University, Chengdu, China; ^3^ Department of Gastroenterology, West China Hospital of Sichuan University, Chengdu, China; ^4^ Department of Oncology, West China (Airport) Hospital, Sichuan University/The First People’s Hospital in Shuangliu District, Chengdu, China

**Keywords:** endothelial to mesenchymal transition, metabolism, glucose metabolic, endothelial, single-cell RNA sequencing

## Abstract

**Background:** Endothelial-to-mesenchymal transition (EndMT) is poorly understood in digestive diseases, and the function of metabolism in EndMT is uncertain.

**Objective:** The goal of this study is to elucidate the role of EndMT in digestive diseases and to describe its metabolic state.

**Method:** The GEO database was used to extract single-cell data in order to discover EndMT subpopulations in digestive organs such as premalignant lesions and cancer of the stomach, intestine, and pancreas.

**Results:** By single-cell RNA sequencing in digestive diseases, we generated a single-cell atlas from tissues of patients spanning a cascade of premalignant lesions and cancer. We next established a single-cell network elucidating the cellular and molecular characteristics of endothelial cells (ECs) across many lesions and identified key genes linked with EndMT in premalignant lesions and cancer lesions. The EndMT activation of a wide variety of metabolic signaling pathways was discovered in ECs, and further study of premalignant lesions and cancer tissue indicated that glucose metabolism increased in premalignant lesions and reached a maximum in cancer tissue. Finally, it was shown that *INSR* and *LDHA* might be used as prognostic markers for developing premalignant lesions to cancer involving glucose metabolism in digestive diseases.

**Conclusion:** For the first time, we discovered EndMT’s role in digestive diseases and described its metabolism, underscoring its crucial role in glucose metabolism in the disease. We found several targets *via* gene screening that are beneficial for predicting premalignant lesions that progress to cancer.

## Introduction

The digestive system includes many organs, including cavity organs such as the oral cavity, esophagus, stomach, intestines, and parenchymal organs such as the liver and pancreas (Hartenstein and Martinez, 2019). It is well established that patients suffering from chronic inflammatory bowel disease (IBD), including ulcerative colitis (UC), or Crohn’s disease (CD) are at an elevated risk of developing colorectal cancer (CRC). As established by mucosal biopsy and standard histology, intestinal cells experience a dysplastic stage prior to adenocarcinoma (Short et al., 2021). Gastric carcinoma is also preceded by premalignant lesions, including chronic atrophic gastritis and intestinal metaplasia for gastric (Zhang et al., 2019). Premalignant pancreatic lesions include benign tumors, intraepithelial neoplasms of the pancreatic ducts, pancreatic neuroendocrine cell tumors, and chronic pancreatitis (Garrido and Djouder, 2021); targeting premalignant lesions is a preventive strategy to prevent malignancies, but the molecular mechanisms of premalignant lesions to malignancy are poorly studied.

Numerous studies have demonstrated that endothelial cells (ECs) can undergo endothelial-to-mesenchymal transition (EndMT), a newly recognized type of cellular transdifferentiation (Xiong et al., 2018). EndMT is a complex biological process in which ECs adopt a mesenchymal phenotype displaying typical mesenchymal cell morphology and functions, including acquiring cellular motility and contractile properties (Gorelova et al., 2021). ECs undergoing EndMT lose EC-specific expression and initiate the expression of mesenchymal cell-specific genes and the production of their encoded proteins (Piera-Velazquez and Jimenez, 2019). Despite intensive investigation, many aspects of EndMT remain to be elucidated. Identifying molecules and regulatory pathways involved in EndMT and discovering specific EndMT inhibitors should provide novel therapeutic approaches for various human disorders mediated by EndMT.

Currently, the metabolic implications of digestive disease and their potential as a target in the management of cancer and its complications are becoming more appreciated. The metabolism of ECs has only recently been recognized as a driving force of angiogenesis (Viallard and Larrivée, 2017). Metabolic pathways, such as glycolysis (Li et al., 2019), fatty acid oxidation (Wong et al., 2017), and glutamine metabolism (Rohlenova et al., 2018) have distinct, essential roles during vessel formation. Numerous studies have shown that EndMT is a tightly regulated active process and that impairment of this regulation could lead to systemic metabolic consequences (Souilhol et al., 2018; Kovacic et al., 2019; Shu et al., 2020). The molecular processes by which digestive ECs contribute to the progression of premalignant lesions to cancer will be explored, emphasizing their effect on the regulation of energy metabolism.

We isolated EndMT subgroups and characterized their metabolic state using tissue samples from digestive diseases with premalignant lesions and cancer in the current public scRNA database. Additionally, we are attempting to uncover key metabolic regulatory genes that may be used to forecast the progression of premalignant lesions to cancer.

## Materials and Methods

### Data Collection

The majority of the scRNA sequencing data were obtained from GEO (https://www.ncbi.nlm.nih.gov/geo/). The majority of the data used in this study are publicly available from published articles: healthy control (esophagus: GEO accession GSE134355, GEO accession GSE160269, and GEO accession GSE173950; stomach: GEO accession GSE134355, and GEO accession GSE134520; https://dna-discovery.stanford.edu/research/datasets/; small intestine: GEO accession GSE134355, and GEO accession GSE134809; colon: GEO accession GSE134355, GEO accession GSE114374, and GEO accession GSE132465; liver: GEO accession GSE136103; pancreas: GEO accession GSE154778 and GEO accession GSE155698; European Genome Archive: EGAS00001004653; spleen: GEO accession GSE134355); chronic atrophic gastritis and atypical hyperplasia (the stomach: GEO accession GSE134520); gastric cancer (stomach: https://dna-discovery.stanford.edu/research/datasets/); inflammatory bowel disease (colon and small intestine: GEO accession GSE134809 and GEO accession GSE114374); intestinal cancer (colon and small intestine: GEO accession 132465); chronic pancreatitis (European Genome Archive: EGAS00001004653); pancreatic cancer (GEO accession GSE155698 and GEO accession GSE154778) (Supplementary Tables S1, S2).

### scRNA-Seq Data Processing, Quality Control, and Data Normalization

We analyzed the scRNA sequencing data using version Seurat v 4.0.2 (Stuart et al., 2019). For initial quality control, we used two criteria: 1) exclusion of individual cells with less than 200 genes and 2) exclusion of individual cells with > 20% mitochondria. Doublet cells exclusion criteria were both high UMI and single cells expressing two or more marker genes at the same clip. We performed LogNormalize() to normalize the gene expression data, used the FindVariableFeatures() function to identify genes whose expression was highly variable between cells, and used ScaleData () to scale the data. Function “RunPCA” was used to find the principal component analysis (PCA).

### Cell Clustering and Dimensional Reduction

Using the default parameters, the harmony package (Korsunsky et al., 2019) was utilized to combine data and eliminate batch effects. Next, the top 20 statistically significant principal components were used in the function “FindNeighbors.” Cells were clustered (cluster resolution = 0.6) by function “FindClusters” and visualized using the UMAP method.

### *In Silico* EC Selection

Endothelial cells were then labeled with classic endothelial cell marker genes (*CLDN5*, *PECAM1*, and *VWF*), and endothelial cells were extracted using Subset (). Cell populations of endothelial cells from seven organs were then pooled together to generate one Seurat object. The Seurat functions FindNeighbors and FindClusters for cell clustering, and UMAP dimensionality reduction was used to generate the final Seurat object.

### Cell Type Identification

The determination of cell type is mainly done by “FindAllMarkers”, where we determine whether a gene is a marker gene by determining the highest expressed gene in each cluster and its significant function, with a minimal fraction of 25% and a log-transformed fold change threshold of 0.25.

### GO and Pathway Enrichment Analyses

Gene Ontology and pathway enrichment analyses (http://geneontology.org/) were performed using the clusterProfiler R package. The annotation Dbi R package org.Hs.eg.db (Carlson M. 2016. org.Hs.eg.db: Genome wide annotation for Human) was used to map gene identifiers. Cluster marker sets and differentially expressed genes were tested individually for overrepresentation, with all expressed/detected genes in each case used as a background control. In each case, the GO gene sets were tested for overrepresentation in cluster markers or differentially expressed genes by computation of enrichment *p* values (the enricher R function, default parameters) from the hypergeometric distribution of total genes in the background gene set, the number of genes within background annotated with the gene set, the size of the gene set, and the number of genes within the cluster marker/differentially expressed genes list annotated with the gene set. Hypergeometric *p* values were adjusted in each case for multiple testing using Benjamini–Hochberg correction as before (clusterProfiler: an R Package for Comparing Biological Themes Among Gene Clusters). The results were visualized as dot plots and umap plots using the R packages clusterProfiler57, enrichplot, and ggplot2.

### Gene Module Score

To score individual cells for pathway activities, we used the R package AUCell (version 1.10.0). First, for each cell, we used an expression matrix to compute gene expression rankings in each cell with the AUCell build rankings function, with default parameters. The canonical pathway database was downloaded from the Broad Institute website, and the canonical pathway gene sets were then used to score each cell where for each gene set and cell, area under the curve (AUC) values were computed (AUCell_calcAUC function) based on gene expression rankings, where AUC values represented the fraction of genes within the top-ranking genes for each cell that are defined as part of the pathway gene set (Aibar et al., 2017). Significant differences between different groups were calculated using the GraphPad Prism 8.0, and the *p* values of less than 0.05 were considered statistically significant.

### Trajectory Analysis

R package monocle3 v1.0.0 was utilized to construct a single cell trajectory analysis (Cao et al., 2019). Cells marked as EndMT and their subsets information were input and constructed as monocle objects.

### Statistical Analysis of Functional Data

We calculated the proportions of cell types in each sample and the t-test was applied. The differentially expressed genes (DEGs) analysis for each cluster was performed using the Wilcoxon rank-sum statistical test. DEGs with a *p*-value less than 0.05 and absolute value natural log fold change greater than 0.25 were used as input for pathway enrichment analyses. When reporting two groups with normal distribution, a two-tailed t-test was applied. We calculated the correlation analysis of the two sample parameters, we used the Spearman correlation coefficient analysis on the log-transformed. The *p* values of less than 0.05 were considered statistically significant. All figures of functional data show the mean ± standard error of the mean (SEM). All statistical analyses were carried out using GraphPad Prism 8.0, or R.

## Results

### Metabolism of Endothelial Cells in Normal Digestive Organs

**FIGURE 1:**
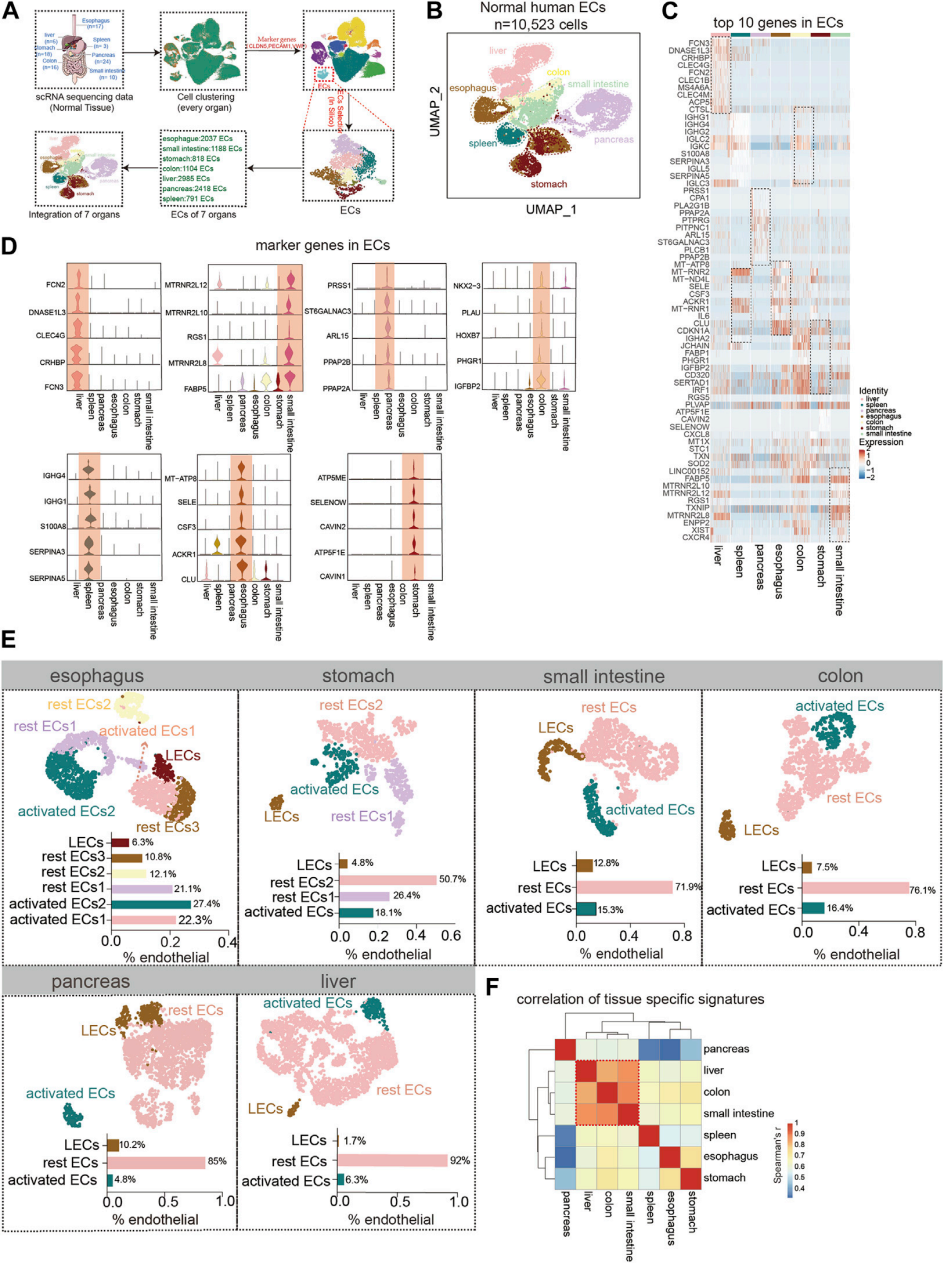
Tissue-specific heterogeneity of endothelial cells (ECs). **(A)** Scheme of *in silico*-selected ECs in this study design. **(B)** Uniform manifold approximation and projection (UMAP) representation of the different digestive tissues from a cell dataset of 10,523 ECs from 93 normal humans (esophagus (*n* = 2,037 cells), stomach (*n* = 818 cells), small intestine (*n* = 1,188 cells), colon (*n* = 1,104 cells), liver (*n* = 2,985 cells), pancreas (*n* = 2,418 cells), and spleen (*n* = 791 cells)). Different colors coded to identify the tissue type (see also Supplementary Table S1). **(C)** Heat map of the top 10 marker genes expressions for each ECs. Color scale: red, high expression; blue, low expression. **(D)** Violin plot showing the high expression of endothelial marker genes in different tissues. **(E)** UMAP plot of esophageal EC subpopulation analysis with cell types classified as activated EC1 (22.3%), activated EC2 (27.4%), rest EC1 (21.1%), rest EC2 (12.1%), rest EC3 (10.8%), and lymphatic ECs (6.3%). The stomach ECs: activated ECs (18.1%), rest EC1 (26.4%), rest EC2 (50.7%), and lymphatic ECs (4.8%). The small intestinal ECs: activated ECs (15.3%), rest ECs (71.9%), and lymphatic ECs (12.8%). The colonic ECs: activated ECs (16.4%), rest ECs (76.1%), and lymphatic ECs (7.5%). The pancreatic ECs: activated ECs (4.8%), rest ECs (85%), and lymphatics ECs (10.2%). The hepatic ECs: activated ECs (6.3%), rest ECs (92%), and lymphatics ECs (1.7%). **(F)** Heat map showing the correlation analysis of gene expression in subpopulations of ECs in each organ.

The human digestive system is divided into hollow organs and parenchymal organs. We collected single-cell data about ECs of the healthy esophagus, stomach, intestine, pancreas, and liver (Figures 1A,B, Supplementary Table S1). The top 10 genes in the ECs of each normal digestive organ were demonstrated using heat maps (Figure 1C). Also, by analysis, we defined 5 marker genes for its different organs (Figure 1D). In the next step, subgroups of endothelial cells from each organ were identified. Using the respective marker genes, they were classified into activated ECs (marker genes: *ACKR1*, *SELE*, and *SELP*), lymphatic endothelial cells (LECs) (marker genes: *LYVE1*, *PDPN*, and *PROX1*), and rest ECs. For the esophagus, the highest percentage of activated ECs was almost 50%, followed by an intermediate percentage of rest ECs and the lowest percentage of LECs. For gastric ECs, the most significant proportion of rest endothelial cells (ECs) was 77 percent, followed by a lower percentage of activated ECs (about 18%), and the lowest percentage of LECs (4.8 percent). The ECs of the small intestine, colon, and stomach share a similar expression profile. Pancreatic ECs contained a larger percentage of resting ECs (about 85%), followed by LECs (10%), and activated ECs (4.8%). The liver has the lowest proportion of LECs, less than 2%, but the highest proportion of rest ECs, 92% (Figure 1E). Finally, we identified the overall Spearman’s correlation of ECs in each digestive organ and found that the liver, colon, and small intestine correlated the most, and the pancreas correlated the least with all other digestive ECs (Figure 1F).

**FIGURE 2:**
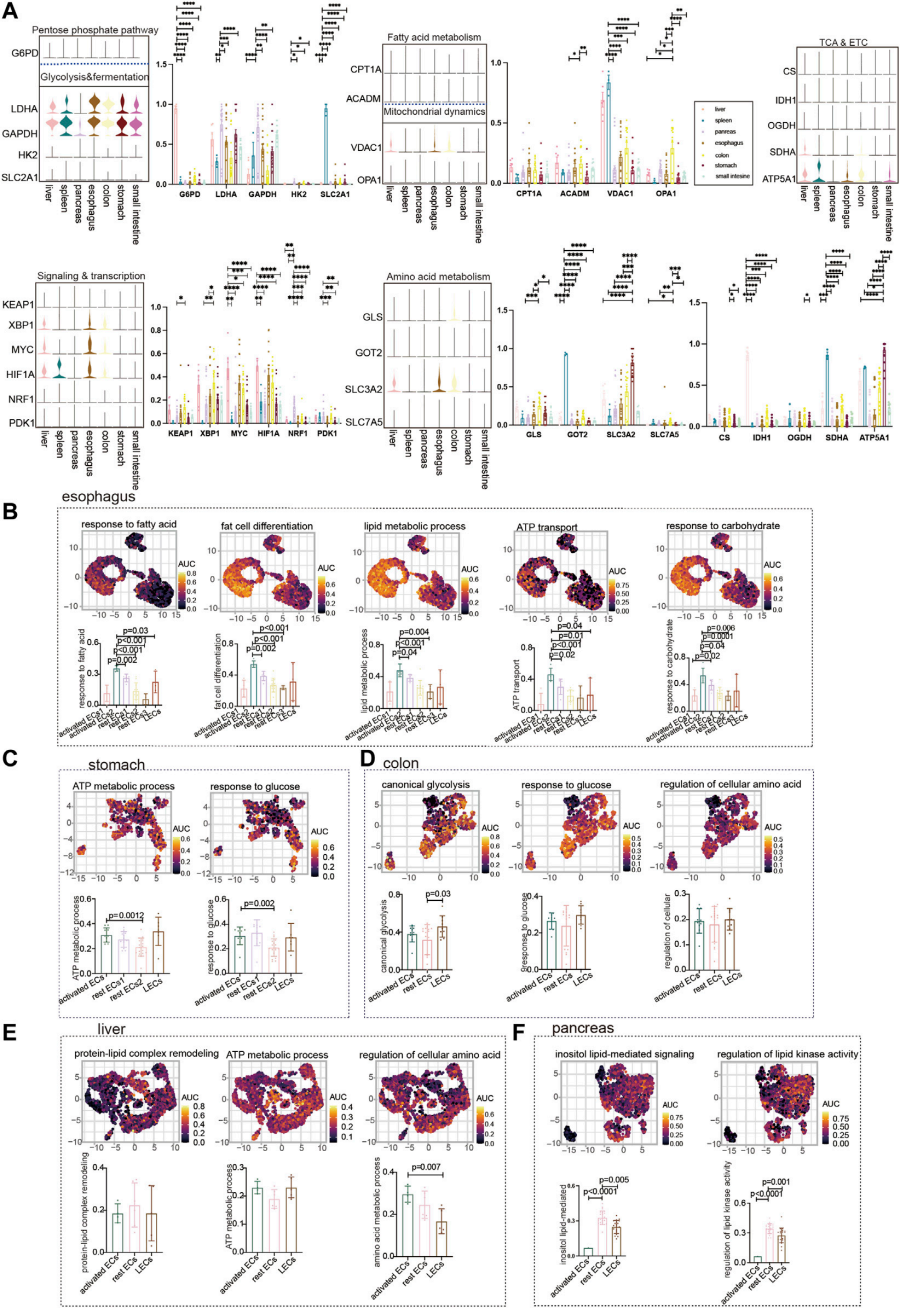
Metabolism of ECs in normal digestive organs. **(A)** Violin plot demonstrating the expression of metabolic regulators in ECs of different tissues. Statistical analysis revealed the proportion of cells positive for metabolic regulators in ECs of different tissues. **(B)** GO analyzed the enrichment of esophageal EC marker genes to metabolism-related signaling pathways (“response to fatty acid,” “fat cell differentiation,” “regulation of lipid metabolic process,” “ATP transport,” and “response to carbohydrate”). UMAP shows the distribution of AUC scores of pathway-related gene clusters for individual cells. The statistical analysis revealed that activated ECs were significantly enriched to metabolic gene pathways compared to other cell populations. **(C)** GO analysis of stomach EC marker genes enriched to metabolism-related signaling pathways (“ATP metabolic process,” “response to glucose”). UMAP shows the distribution of AUC scores of pathway-related gene clusters for individual cells. The statistical analysis revealed that activated ECs were significantly enriched in metabolic gene pathways. **(D)** Marker genes of colonic ECs were analyzed by GO to be enriched to metabolism-related signaling pathways (“canonical glycolysis,” “response to glucose,” “regulation of cellular amino acid metabolic process”). UMAP shows the distribution of AUC scores of pathway-associated gene clusters for individual cells. **(E)** GO analysis of hepatic EC marker genes enriched to metabolism-related signaling pathways (“ATP metabolic process,” “protein–lipid complex remodeling,” and “regulation of cellular amino acid metabolic process”). UMAP shows the distribution of AUC scores of pathway-related gene clusters for individual cells. **(F)** GO analysis of pancreatic EC marker genes enriched for metabolism-related signaling pathways (“inositol lipid-mediated signaling” and “regulation of lipid kinase activity”). UMAP shows the distribution of AUC scores of pathway-related gene clusters in individual cells. The statistical analysis revealed that LECs were significantly enriched in metabolic gene pathways.

We showed the expression of common metabolic genes in normal ECs of different tissues in the digestive system (Figure 2A). Furthermore, we used the GO analysis to describe the metabolic profile of normal gastrointestinal ECs. The metabolism of the esophagus is different from that of other organs. It is mainly activated by the endothelial metabolic response to fatty acids, lipids, and carbohydrates. The highest AUC score was achieved by activated ECs2 enrichment among the aforementioned metabolic pathways (Figure 2B). The metabolism in the stomach was enriched for ATP and glycolysis metabolism. As with the esophagus, the stomach’s metabolism is dominated by activated ECs and LECs to a lesser degree (Figure 2C). No active metabolic gene sets were found in the small intestine. The colon is enriched for glycolysis and amino acid metabolism. The metabolic expression trends of LECs, rest ECs, and activated ECs of the colon were consistent (Figure 2D). The liver mainly responds to endothelial amino acid metabolism. Using the statistical ACU score, hepatic regulation of cellular amino acid was higher in the expression of activated ECs (Figure 2E). The pancreas is mainly lipid metabolized. Interestingly, the metabolic pathways of rest ECs are most highly expressed in the pancreas (Figure 2F).

### Glucose Metabolism Status of Endothelium-to-Mesenchymal Transition in Gastric Premalignant Lesions and Gastric Cancer

**FIGURE 3:**
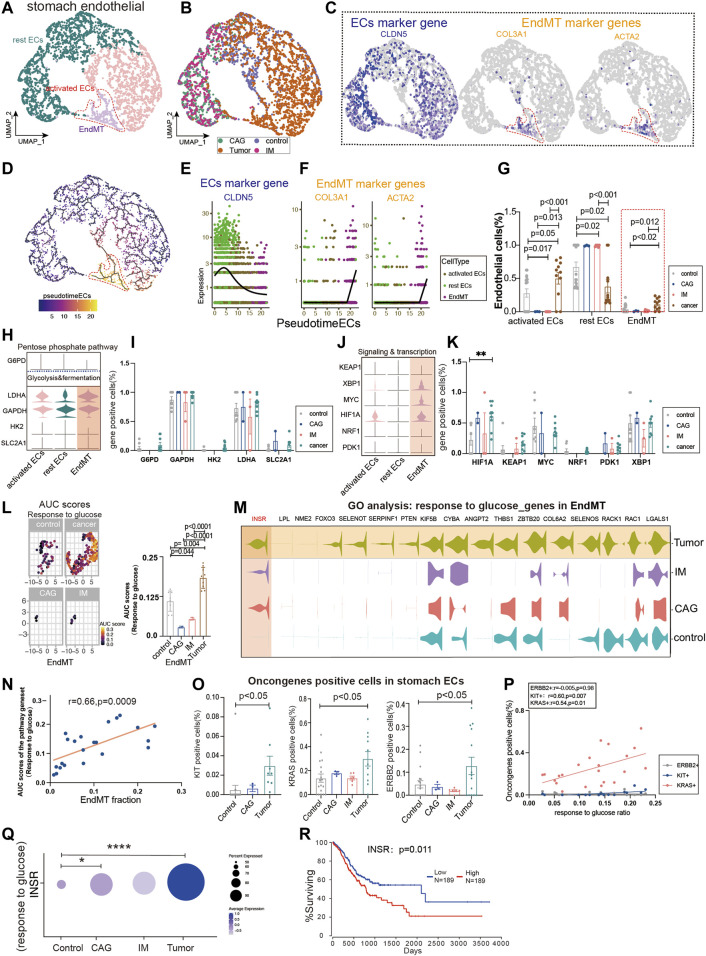
Glucose metabolism status of endothelial-to-mesenchymal transition (EndMT) in gastric premalignant lesions and gastric cancer. **(A, B)** UMAP of 3,558 stomach ECs from 18 control samples, 3 CAG, 6 IM, and 11 gastric cancer. Each dot represents a single cell, and cells are labeled by cell type. UMAP demonstrates the cellular subpopulations of the stomach ECs, including EndMT, activated ECs, and rest ECs (see also Supplementary Table S2,S3). **(C)** UMAP plots showing EC-specific marker genes (*CLDN5*) and mesenchymal cell-specific marker genes (*COL3A1* and *ACTA2*). **(D)** Pseudo-temporal analysis showing the evolutionary trend of ECs as “rest EC-activated EC-EndMT.” **(E, F)** Temporal variation of EC marker genes (*CLDN5*), EndMT marker genes (*COL3A1* and *ACTA2*). **(G)** Cell proportions of each cell subpopulation of ECs in healthy individuals and patients with CAG, IM, and gastric cancer, and cell proportions of EndMT were significantly higher in gastric cancer patients. **(H–K)** Violin plot demonstrating the expression of metabolic regulators in stomach ECs. *LDHA* and *GAPDH* in the glycolysis and fermentation metabolic pathway had the highest expression in EndMT, in signaling and transcription, genes such as *HIF1A*, *MYC*, and *XBP1*, had the highest expression in EndMT. **(L, M)** In EndMT, gastric cancer patients were significantly enriched to glucose metabolic-related signaling pathways: “response to glucose”; UMAP shows the distribution of AUC scores of pathway-related gene clusters for individual cells, with statistically significant AUC scores between gastric cancer and normal control/CAG/IM groups. Violin plot demonstrating the expression of glucose metabolic-related genes in stomach ECs. **(N)** Correlation analysis revealed that the EndMT fraction in stomach ECs was positively correlated with the AUC score of the glucose metabolic pathway gene set. **(O)** Statistical analysis revealed that the proportion of cells positive for oncogenes *KRAS*, *KIT*, and *ERBB2* genes was significantly higher in gastric cancer patients. **(P)** Correlation analysis revealed that the AUC score of the glucose metabolic pathway gene set was positively correlated with the proportion of cells positive for oncogenes *KRAS* and *KIT*. **(Q)** Dot plot showing that INSR, an essential gene in the response to the glucose pathway, was the most highly expressed in gastric cancer, followed by CAG, and control. **(R)** Survival analysis showing that the higher the INSR, the lower the survival rate, and there was a statistical difference.

We extracted ECs from four diseases (control, chronic atrophic gastritis (CAG), intestinal metaplasia (IM), and tumor) to observe the expression of different subpopulations of ECs. Gastric Ecs are divided into several subgroups: lymphatic Ecs, activated Ecs, rest Ecs, and EndMT (Figures 3A,B, Supplementary Figure S1A; Supplementary Tables S2, S3). The marker genes that define all gastric endothelium are *CLDN5*, *ACTA2*, and *COL3A1* are marker genes of gastric EndMT (Figure 3C). Furthermore, the expression trend of EndMT was identified using package monocle3, suggesting a transition from rest ECs to activated ECs and finally to EndMT in the gastric endothelium (Figure 3D). *CLDN5* was seen to decrease in expression with the endothelial transition to EndMT; *ACTA2* and *COL3A1* were upregulated with EndMT expression upshift (Figures 3E,F). Activated ECs and EndMT had the highest proportions in tumors (Figure 3G). Furthermore, we investigated the expression of key regulatory genes of common metabolic pathways, and *LDHA* and *GAPDH* in glycolysis and fermentation metabolic pathways had the highest expression in EndMT (Figures 3H,I). Also, in signaling and transcription, genes such as *HIF1A*, *MYC*, and *XBP1* had the highest expression in EndMT (Figures 3J,K). The core genes of fatty acid metabolism, TCA and, ETC, and amino acid metabolism metabolic pathways also expressed highest mainly in EndMT (Supplementary Figure S2A). EndMT metabolism was significantly higher in gastric cancer than in healthy controls, and the main increased metabolisms were glycosaminoglycan catabolic process, and amino acid metabolism (Supplementary Figures S2B,C). Finally, the expression of metabolic signaling pathways in ECs in four diseases was identified. First, response to glucose was highest in tumor and lowest in CAG (Figure 3L–M). EndMT was correlated with response to glucose with r = 0.66, *p* = 0.0009(Figure 3N). Metabolic-related signaling pathways such as nucleoside triphosphate metabolic, response to carbohydrate, response to an amino acid, and ATP metabolic process were enriched, and the expression trend was still dominated by high gastric cancer (Supplementary Figures S2D–G). After screening for oncogenes or suppressor genes, *KIT*, *KRAS*, and *ERBB2* were significantly higher in gastric cancer ECs (Figure 3O). Oncogenes were strongly correlated with glucose metabolism, nucleoside triphosphate, metabolic ATP metabolism, response to an amino acid, and response to carbohydrate (Figure 3P; Supplementary Figure S2H). Using the metabolic pathway genes for glucose metabolism, we found that INSR expression was consistently elevated from premalignant lesions to gastric cancer, while high expression of INSR predicted lower survival rates (Figures 3Q,R).

### Glucose Metabolic Status of Endothelium-to-Mesenchymal Transition in Inflammatory Bowel Disease and Colon Cancer

**FIGURE 4:**
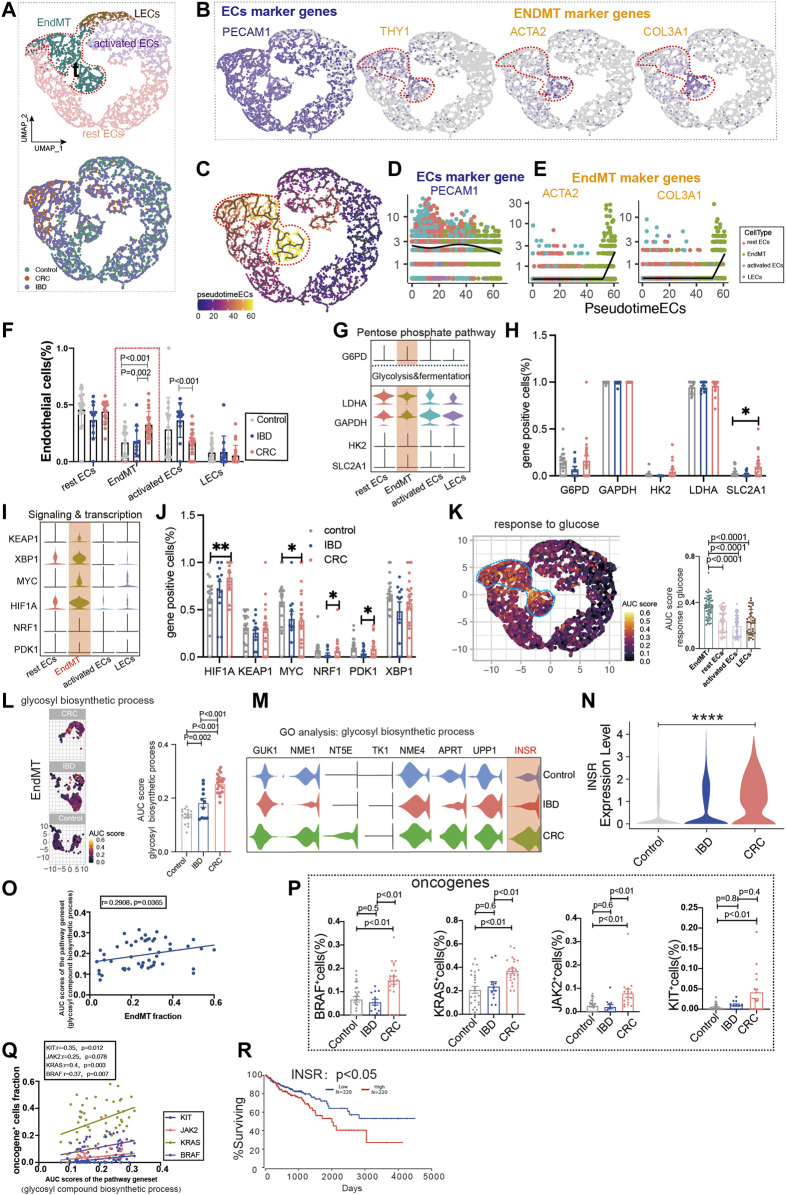
Glucose metabolic status of EndMT in IBD and colon cancer. **(A)** UMAP of 5,356 intestinal ECs from 26 control samples, 12 inflammatory bowel disease (IBD), 22 colorectal cancer (CRC). Each dot represents a single cell, and cells are labeled by cell type. UMAP demonstrates the cellular subpopulations of the gut ECs, including EndMT, activated ECs, rest ECs, and lymphatic ECs. (See also Supplementary Table S2, S3). **(B)** UMAP plots showing EC-specific marker genes (*PECAM1*) and EndMT marker genes (*THY1*, *ACTA2*, and *COL3A1*). **(C)** Pseudo-temporal analysis shows the evolutionary trend of ECs as “rest EC-activated EC-EndMT.” **(D, E)** Temporal variation of EC marker genes (*PECAM1*) and EndMT marker genes (*THY1*, *ACTA2*, and *COL3A1*). **(F)** Cell proportions of each cell subpopulation of ECs in healthy individuals, IBD and CRC, and cell proportions of EndMT were significantly higher in CRC patients. **(G–J)** Violin plot showing the expression of metabolic regulators in ECs. The statistical analysis revealed the proportion of cells positive for metabolic regulators. **(K)** GO analysis shows the enrichment of intestinal EndMT cell marker genes to glucose metabolic-related signaling pathways: “response to glucose.” UMAP shows the distribution of AUC scores of pathway-related gene clusters for individual cells. The statistical analysis revealed that EndMT was significantly enriched to metabolic gene pathways compared to other cell populations. Violin plot demonstrating the expression of glucose metabolic-related genes in ECs. **(L)** In EndMT, patients with CRC were significantly enriched in metabolic-related signaling pathways: “glycosyl compound biosynthetic.” UMAP shows the pathway-related gene cluster AUC of individual cell score distribution, with statistically significant AUC scores between CRC and normal controls/IBD. **(M, N)** Violin plot showing that INSR, a key gene in the glucose metabolic-related signaling pathways, was the most highly expressed in CRC. **(O)** Correlation analysis revealed that EndMT was positively correlated with glucose metabolic pathway gene set AUC score. **(P)** Statistical analysis revealed that the proportion of cells positive for *KRAS*, *KIT*, *BRAF*, and *JAK2* genes was significantly higher in patients with CRC. **(Q)** Correlation analysis revealed that the glucose metabolic pathway gene set AUC score was positively correlated with the proportion of cells positive for *KRAS*, *KIT*, *BRAF*, and *JAK2* genes. **(R)** Survival analysis showing that the higher the INSR, the lower the survival rate, and there was a statistical difference.

Colon cancer is one of the major outcomes of IBD. By analyzing ECs from healthy controls, IBD, and colon cancer, we classified ECs into lymphatic ECs, rest ECs, activated ECs, and EndMT (Figure 4A; Supplementary Figure S1B; Supplementary Table S2, S3). The marker genes of ECs were PECAM1, and the marker genes of EndMT were *ACTA2* and *COL3A1*, and *THY1*, respectively (Figure 4B). Also, we used the trajectory analysis to see the conversion of EndMT (Figure 4C). The expression of PECAM1, a marker gene for total intestinal endothelial cells, was decreased in the transformation of EndMT with a concomitant increase in *ACTA2* and *COL3A1* (Figures 4D,E). The proportion of EndMT in colon cancer was higher than IBD and control, and IBD was higher than control (Figure 4F). Glycolysis and fermentation of major metabolic genes were highly expressed inside EndMT, and *SLC2A1* was highest expressed gene in colon cancer (Figures 4G,H). We also examined the expression of genes and transcription factors in key metabolic signaling pathways across the subpopulation, the most abundant genes in colon cancer were *PDK1*, *NRF1*, *MYC*, and *HIF1A* (Figures 4I,J). TCA and ETC, amino acid metabolism, and fatty acid metabolism were also highest in EndMT (Supplementary Figure S3A). Response to glucose metabolism, fatty acid metabolism, and response to amino acids was highly expressed in EndMT (Figure 4K; Supplementary Figure S3B). For EndMT, the expression of glycosyl biosynthetic process was highest in colon cancer, followed by IBD, and lowest in control (Figures 4L,M). INSR was progressively upregulated in IBD and colon cancer (Figure 4N). EndMT is positively correlated with the glycosyl biosynthetic process (Figure 4O). In colon cancer, ATP and lipid metabolism were elevated (Supplementary Figure S3C). After screening for oncogenes or suppressor genes, *KIT*, *KRAS*, *BRAF*, and *JAK2* were significantly higher in colon cancer ECs (Figure 4P). Oncogene-positive cells correlate with metabolic gene sets (Supplementary Figure S3D). High expression of INSR, a vital gene of the glucose metabolic pathway, has a lower survival rate in colon cancer (Figure 4R).

### Glucose Metabolic Status of EndMT in Chronic Pancreatitis and Pancreatic Cancer

**FIGURE 5:**
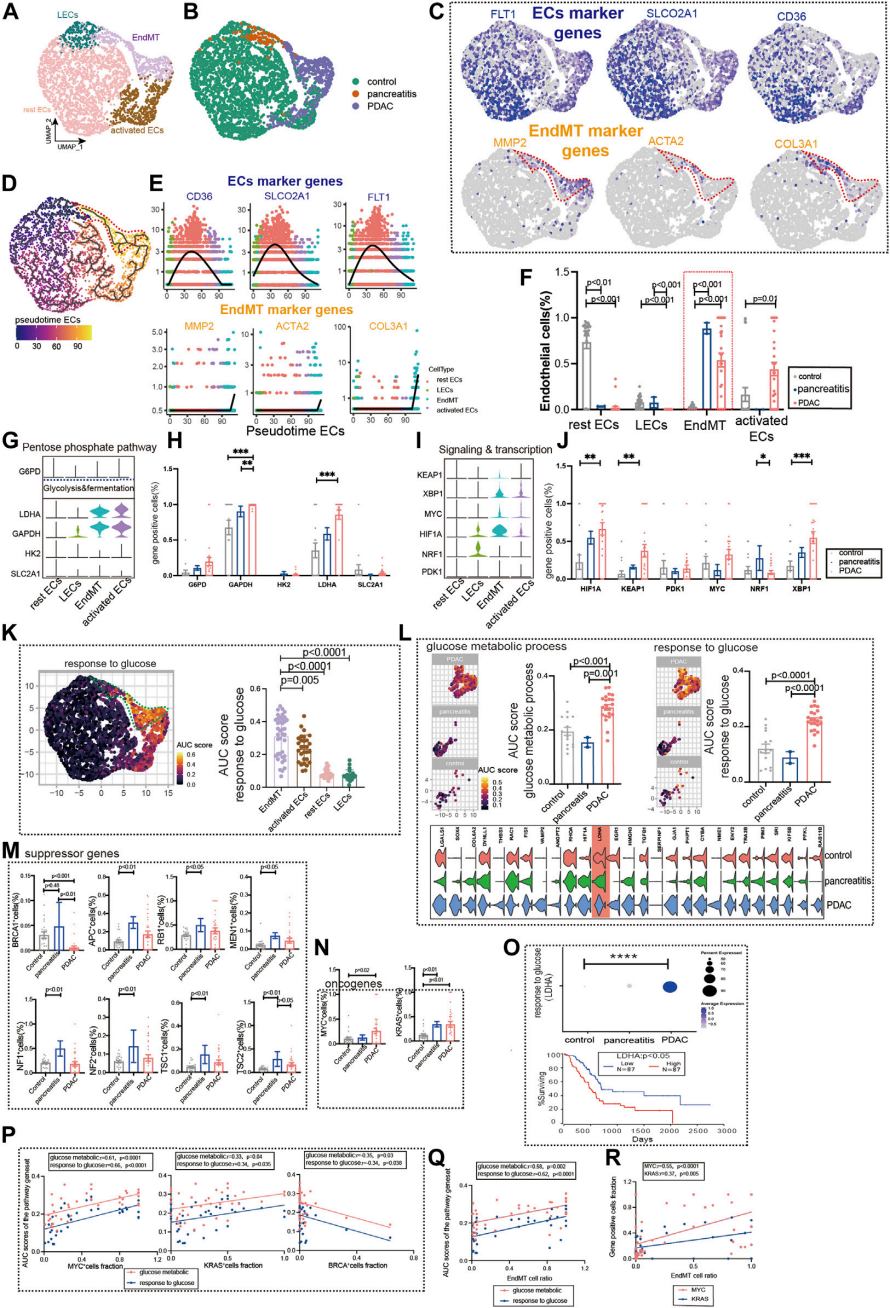
Glucose metabolic status of EndMT in chronic pancreatitis and pancreatic cancer. **(A, B)** UMAP of 2,980 pancreatic ECs, from 21 control samples, 2 chronic pancreatitis, and 30 pancreas cancer. Each dot represents a single cell, and cells are labeled by cell type. UMAP demonstrates the cellular subpopulations of the pancreatic ECs, including EndMT, activated ECs, rest ECs, and lymphatic ECs (see also Supplementary Table S2, S3). **(C)** UMAP plots show EC-specific marker genes (*FLT1*, *SLCO2A1*, and *CD36*) and EndMT-specific marker genes (*MMP2*, *ACTA2*, and *COL3A1*). **(D)** Pseudotime analysis showing the evolutionary trend of ECs as “rest EC-activated EC-EndMT.” **(E)** Temporal variation of EndMT marker genes (*MMP2*, *ACTA2*, and *COL3A1*), EC marker genes (*FLT1*, *SLCO2A1*, and *CD36*). **(F)** Cell proportions of each cell subpopulation of ECs in healthy individuals, chronic pancreatitis, and pancreatic cancer patients, and cell proportions of EndMT were significantly higher in chronic pancreatitis and pancreatic cancer patients. **(G–J)** Violin plot demonstrating the expression of metabolic regulators in ECs. *LDHA* and *GAPDH* in the glycolysis and fermentation metabolic pathway had the highest expression in EndMT. The statistical analysis revealed that the proportion of cells positive for *LDHA* and *GAPDH* in EndMT was significantly higher for pancreatic cancer patients. In signaling and transcription, genes such as *HIF1A*, *XBP1*, and *KEAP1* had the highest expression in EndMT. The statistical analysis revealed that the proportion of cells positive for *HIF1A*, *XBP1*, and *KEAP1* in EndMT was significantly higher for pancreatic cancer patients. **(K)** GO analysis shows the enrichment of pancreatic EndMT cell marker genes to glucose metabolism-related signaling pathways (“ response to glucose ”). UMAP shows the distribution of AUC scores of pathway-related gene clusters for individual cells. **(L)** In EndMT, pancreatic cancer patients were significantly enriched to glucose metabolic-related signaling pathways: “ response to glucose ” and “glucose metabolic process.” UMAP shows the distribution of AUC scores of pathway-related gene clusters for individual cells, with statistically significant AUC scores between pancreatic cancer and normal controls/chronic pancreatitis. **(M)** Statistical analysis revealed the proportion of cells positive for suppressor genes *BRCA* was lower in pancreatic cancer patients. **(N)** Statistical analysis revealed that the proportion of cells positive for oncogenes *KRAS* and *MYC* genes was significantly higher in pancreatic cancer patients. **(O)** Dot plot showing that a key gene *LDHA* in response to the glucose pathway was the most highly expressed in pancreatic cancer. The survival analysis showing that the higher *LDHA* was associated with a lower survival rate and was statistically different. **(P)** Correlation analysis revealed that the AUC score of metabolic-related signaling pathways was also positively correlated with the proportion of cells positive for oncogenes *KRAS* and *MYC* genes and negatively correlated with the proportion of cells positive for suppressor genes *BRCA* (color scale: red: glucose metabolic; blue: response to glucose). **(Q)** Correlation analysis revealed that EndMT was positively correlated with the AUC score of the metabolic pathway gene set (color scale: red: glucose metabolic; blue: response to glucose). **(R)** Correlation analysis revealed that EndMT was positively correlated with the fraction of oncogenes *KRAS-* and *MYC*-positive cells.

Chronic pancreatitis (CP) has been linked to an increased risk of pancreatic cancer in several studies (Malka et al., 2002). Subpopulations of the pancreatic ECs, including EndMT, activated ECs, rest ECs, lymphatic ECs (Figures 5A,B, Supplementary Figure S1C, Supplementary Table S2, S3). Pancreatic ECs suggest *FLT1*, *SLCO2A1*, and *CD36* as genes for endothelial development, which are reduced in late endothelial development. Marker genes of EndMT (*MMP2*, *ACTA2*, and *COL3A*) have lower expression in early development and higher expression in late development (Figures 5C–E). The trajectory analysis suggests the beginning of the endothelium from the lymphatic duct, with EndMT as the end (Figure 5D). The EndMT ratio was higher in pancreatic cancer than in healthy control patients (Figure 5F). We also compared the relationship between genes of common metabolic pathways and in different subgroups of the pancreas (Figures 5G–J, Supplementary Figure S4A).

Furthermore, we analyzed the metabolic changes among several subgroups. Mesenchyme plays an essential role in glucose metabolism, LDL metabolism, ATP metabolism, and amino acid metabolism (Figure 5K, Supplementary Figure S4B). EndMT of pancreatic ductal adenocarcinoma (PDAC) was also found to have the highest expression in the glucose metabolism, ATP metabolic process, and LDL metabolism (Figure5L, Supplementary Figure S4C). By screening for common oncogenes and suppressor genes, endothelium *KRAS-* and *MYC*-positive genes were enhanced in pancreatic cancer expression (Figure 5N). We found that *BRCA*-positive genes were significantly lower in pancreatic cancer ECs (Figure 5M). Survival in pancreatic cancer can indeed be predicted by the glucose metabolism key gene *LDHA* (Figure 5O). Oncogenes (*KRAS* and *MYC*) were strongly correlated with glucose metabolism pathways upregulated by EndMT, whereas the suppressor gene (*BRCA*) was negatively correlated (Figure 5P). In addition, EndMT correlates with glucose metabolic gene sets and oncogenes (Figures 5Q,R, Supplementary Figure S5D).

## Discussion

This study revealed novel insights into various aspects. First, we identified the top 5 marker genes that were highly enriched in most ECs from the esophagus, stomach, intestine, liver, and pancreas; second, based on the discovery of partially overlapping transcriptome features and common inferred biological features, we found similar expression profiles in the stomach, colon, and small intestine, but specific expression profiles in the pancreas; third, in the search of vascular endothelial bed-specific markers, we found that markers for arteries, veins and lymphatics were shared by most tissues, suggesting phenotypic conservation of these vascular bed markers in different tissues. These results also coincide with the results in mice (Kalucka et al., 2020).

Fourth, ECs have been shown to be predictive of cellular metabolism in some studies (Alonso-Herranz et al., 2020; Shu et al., 2020; Takagaki et al., 2020). The metabolism of normal ECs of the human digestive system is currently not described in the literature, and we describe the transcriptional expression levels of genes from the common metabolome at the single-cell level. Proliferating ECs rely primarily on glycolysis metabolism in normal ECs (De Bock et al., 2013). As readout from our scRNA-seq analysis, our study exhibited heterogeneity in different tissues in terms of the rest ECs, activated ECs, and lymphatic ECs metabolism in the normal digestive system. Our study also found the expression of key rate-limiting enzymes in glucose metabolism in normal ECs.

Fifth, our study is also comparing the metabolic profile changes between premalignancy lesions and cancer, which provides ideas for the prevention and treatment of premalignancy. ECs of the digestive disease have been less studied in premalignant lesions, and it is conventionally believed that the endothelial function of the digestive system is mainly blood transport. However, the role of the ECs in digestive disease goes far beyond the protection of blood transport (DeLeve and Maretti-Mira, 2017; Xu et al., 2019; Kalucka et al., 2020). The previous study has established that elevated hyperglycemia (HG) damages endothelial cells (ECs) through EndMT and EMT (Ahn et al., 2017; Youssef and Nieto, 2020). Our study also concluded from our analysis that endothelial metabolism plays a vital role in the progression of premalignancy lesions and cancer in digestive diseases. We displayed that glucose metabolism encompasses a diverse range of metabolic pathways after evaluating the metabolic features of precancerous lesions and cancerous tissues. To begin, premalignant lesions tissues have a high concentration of glycolytic signaling pathways, and again, as tissue heterogeneity develops, glycolytic signaling pathways increase as well. Finally, we discovered critical prognostic genes related to glycolytic signaling pathways expressed in premalignant lesions and rise quickly in cancerous tissues.

Our study also has some shortcomings. 1) the sample size of early cancer patients was too small, and the weight of statistics can be unbalanced; 2) the samples lacked matching clinical characters, and correlations with clinical features were difficult to describe; and 3) we did not count whether the endothelium of mice had EndMT expression and whether the expression trend was consistent with that of humans.

The metabolic status of ECs of the gastrointestinal tract has also been less studied for the early prediction of cancer. Mesenchymal transition of ECs is a necessary process for the progression of cancer and has an important role in the current prediction of cancer. Furthermore, our study found that genes regulating glucose metabolism in mesenchyme-related genes also predict tumorigenesis. Finally, we established the EndMT-metabolic axis of the digestive disease, using EndMT to predict whether premalignant lesions transform into cancer, and established the relationship between EndMT and glucose metabolism, suggesting that activation of metabolic pathways is also associated with cancer.

## Data Availability

The original contributions presented in the study are included in the article/Supplementary Material; further inquiries can be directed to the corresponding author.
