# Supplementary figures and images for: Single Cell Meta-Analysis of Endothelial to Mesenchymal Transition (EndMT) in Glucose Metabolism of the Digestive Diseases
Source: Front Mol Biosci. 2022 Jun 8;9:866408. doi: 10.3389/fmolb.2022.866408 (PMC9216733; doi:10.3389/fmolb.2022.866408)

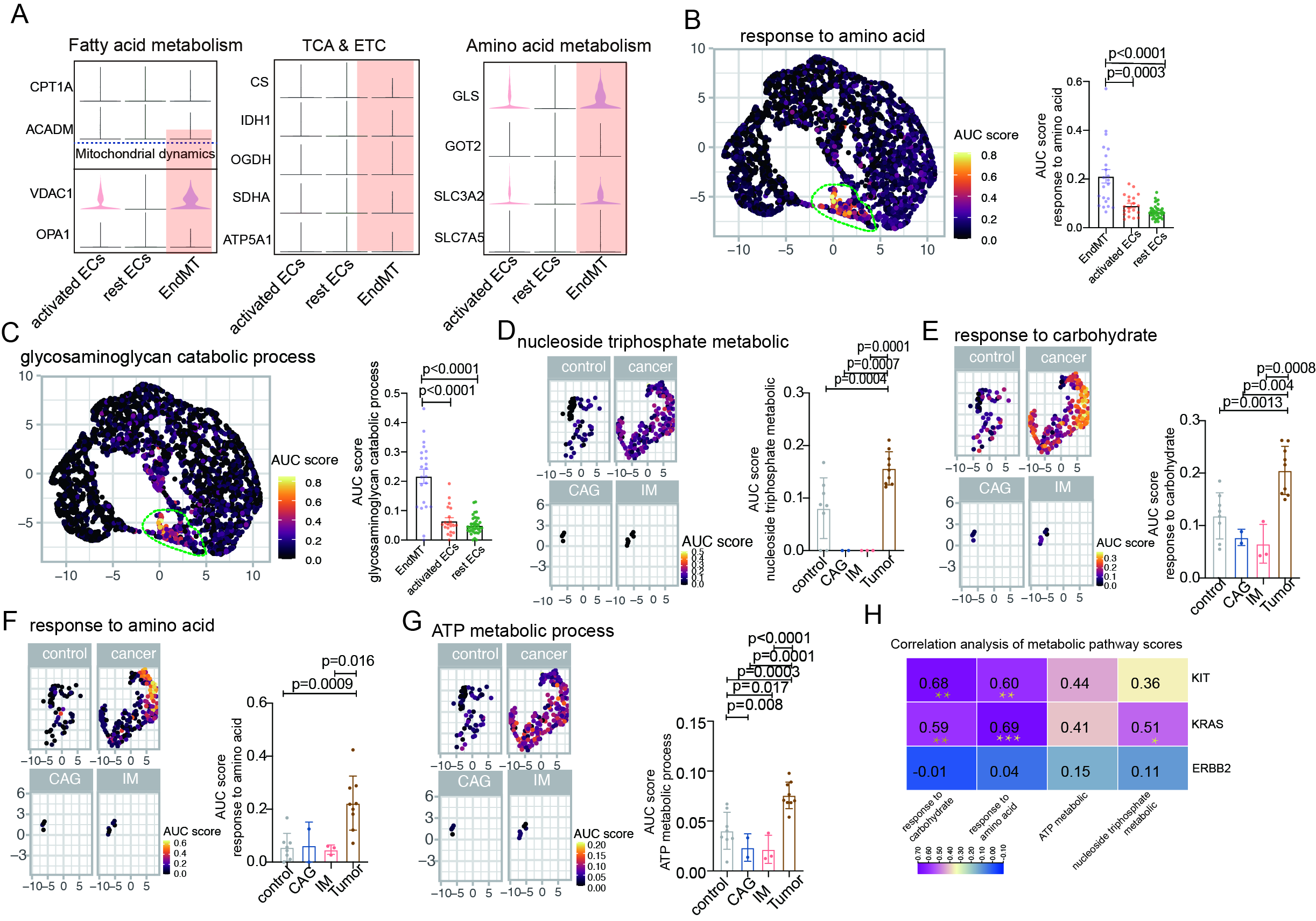

Supplement: Supplementary file 1 [file DataSheet1.zip › Supplementary material/sFigure/Figure S2.tif]

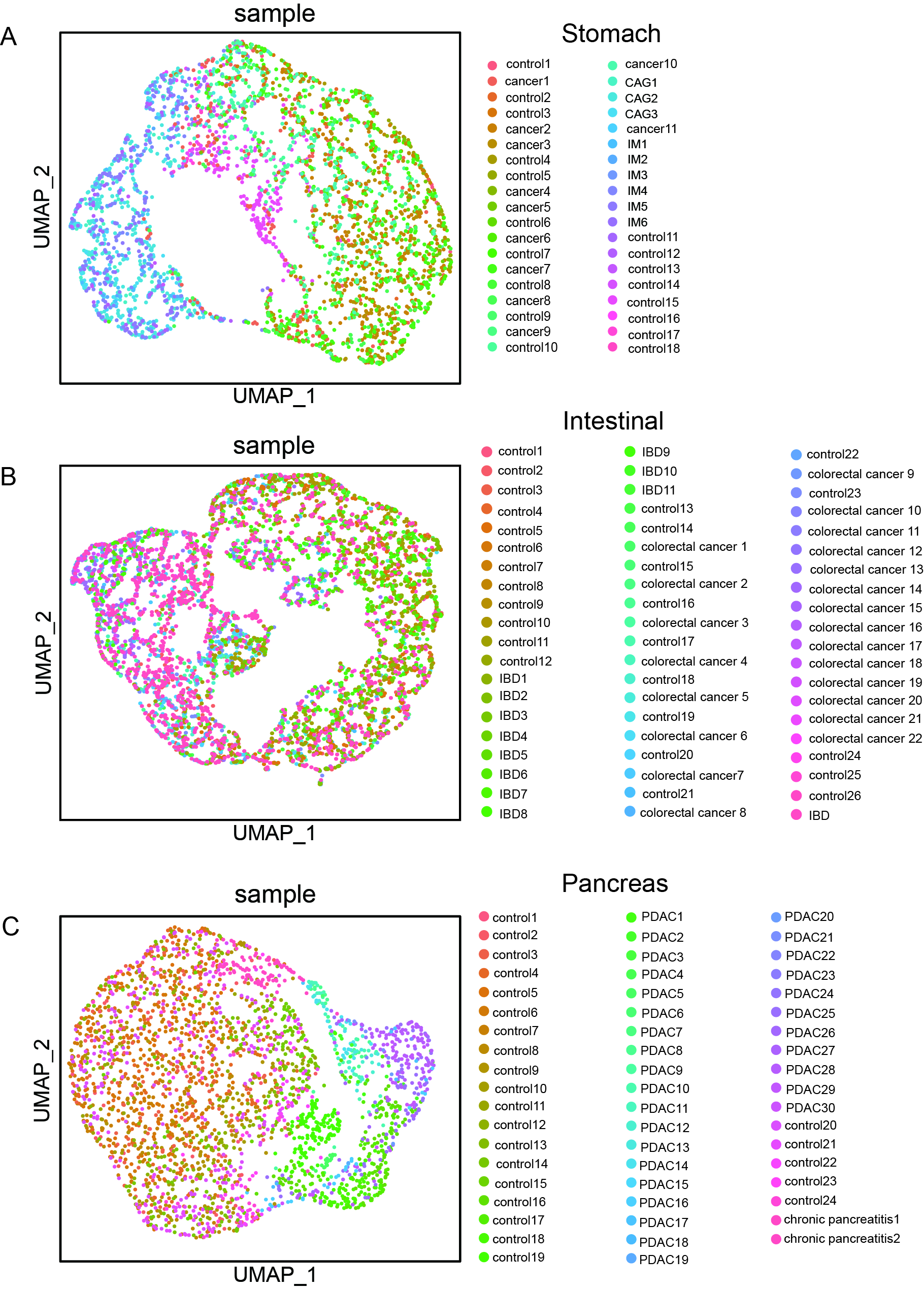

Supplement: Supplementary file 1 [file DataSheet1.zip › Supplementary material/sFigure/Figures S1.tif]

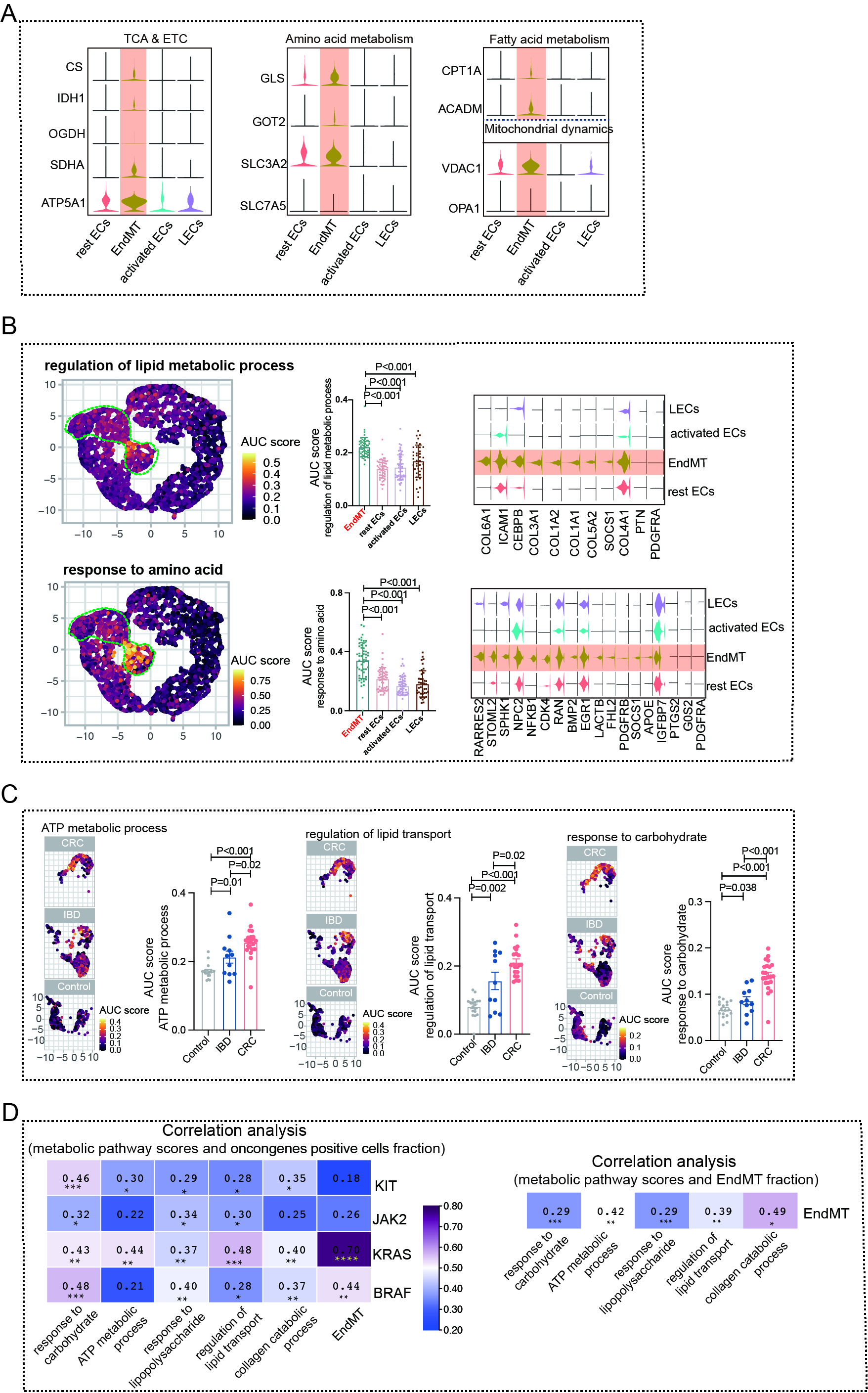

Supplement: Supplementary file 1 [file DataSheet1.zip › Supplementary material/sFigure/Figures S3.tif]

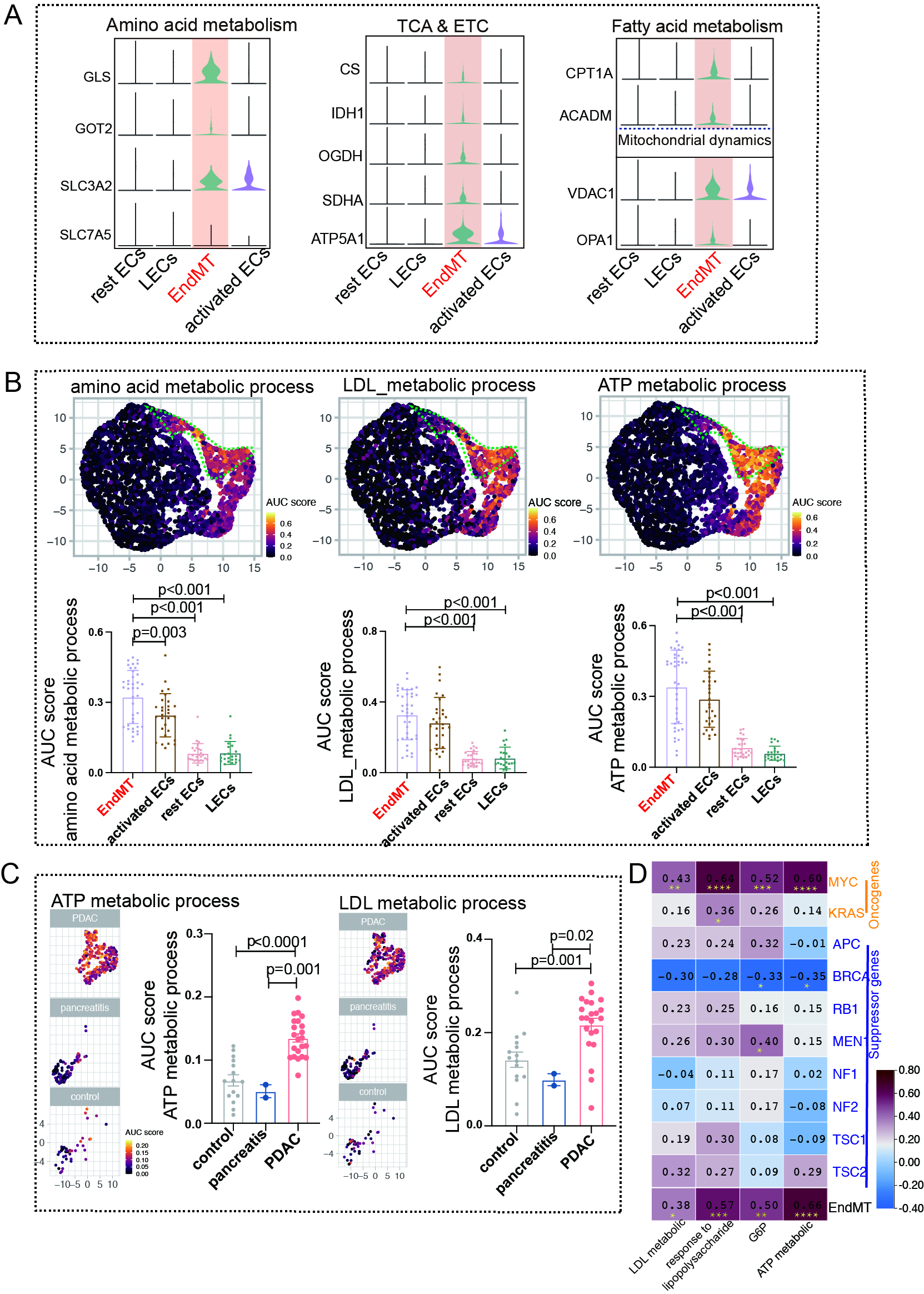

Supplement: Supplementary file 1 [file DataSheet1.zip › Supplementary material/sFigure/Figures S4.tif]
